# Human METTL7B is an alkyl thiol methyltransferase that metabolizes hydrogen sulfide and captopril

**DOI:** 10.1038/s41598-021-84218-5

**Published:** 2021-03-01

**Authors:** Benjamin J. Maldonato, Drake A. Russell, Rheem A. Totah

**Affiliations:** Department of Medicinal Chemistry, University of Washington, 1959 NE Pacific Ave, Box 357610, Seattle, WA 98195 USA

**Keywords:** Enzymes, Transferases

## Abstract

Methylation of alkyl thiols is a biotransformation pathway designed to reduce thiol reactivity and potential toxicity, yet the gene and protein responsible for human alkyl thiol methyltransferase (TMT) activity remain unknown. Here we demonstrate with a range of experimental approaches using cell lines, in vitro systems, and recombinantly expressed enzyme, that human methyltransferase-like protein 7B (METTL7B) catalyzes the transfer of a methyl group from *S-*adenosyl-l-methionine (AdoMet) to hydrogen sulfide (H_2_S) and other exogenous thiol small molecules. *METTL7B* gene modulation experiments, including knockdown in HepG2 cells and overexpression in HeLa cells, directly alter the methylation of the drug captopril, a historic probe substrate for TMT activity. Furthermore, recombinantly expressed and purified wild-type METTL7B methylates several thiol compounds, including H_2_S, 7α-thiospironolactone, l-penicillamine, and captopril, in a time- and concentration-dependent manner. Typical for AdoMet-dependent small molecule methyltransferases, *S*-adenosyl-l-homocysteine (AdoHcy) inhibited METTL7B activity in a competitive fashion. Similarly, mutating a conserved aspartate residue, proposed to anchor AdoMet into the active site, to an alanine (D98A) abolished methylation activity. Endogenous thiols such as glutathione and cysteine, or classic substrates for other known small molecule *S*-, *N*-, and *O*-methyltransferases, were not substrates for METTL7B. Our results confirm, for the first time, that *METTL7B*, a gene implicated in multiple disease states including rheumatoid arthritis and breast cancer, encodes a protein that methylates small molecule alkyl thiols. Identifying the catalytic function of METTL7B will enable future pharmacological research in disease pathophysiology where altered *METTL7B* expression and, potentially H_2_S levels, can disrupt cell growth and redox state.

## Introduction

Thiol methylation in humans is catalyzed by two enzymes, thiopurine methyltransferase (TPMT)^1,2^ and thiol methyltransferase (TMT)^3^. TPMT selectively methylates thiopurine compounds while TMT is selective for aliphatic thiol substrates^4–6^. Both enzymes exhibit highly variable activity in vivo which can lead to toxicity for thiol-containing drugs that undergo methylation, such as azathioprine and the active metabolite of clopidogrel^7–14^. While TPMT has been extensively characterized, TMT is a putative microsomal enzyme that selectively methylates aliphatic thiols, with potential substrates including hydrogen sulfide (H_2_S), captopril, 7α-thiospironolactone, d- and l-penicillamine, and the active metabolites of prasugrel, clopidogrel, and ziprasidone^6,15–20^. To date, and despite numerous attempts, researchers have not successfully identified the microsomal TMT gene or protein^3,21–23^.

Despite an unknown function, *METTL7B* has surfaced in several genetic studies linking its upregulation to multiple disease states^24,25^. Specifically, *METTL7B* expression is significantly altered in kidney disease, acute respiratory distress syndrome, and numerous cancers, including breast, non-small cell lung, thyroid, and ovarian^24–30^. In non-small cell lung cancer, upregulated *METTL7B* contributes to tumorigenesis and progression by regulating cell cycle progression, suggesting that it may be a potential therapeutic target. In fact, silencing *METTL7B* gene expression in vitro and in vivo reduced tumor growth and progression^30^. Additionally, *METTL7B* expression appears to be responsive to inflammation signaling pathways via JAK1^31,32^. Gene expression is also sensitive to cellular redox state and is associated with individual response to certain chemotherapeutics^33,34^. Further investigation is required to determine the exact role of METTL7B in human health and disease.

Human *METTL7B* is predicted to encode a full-length protein with a molecular weight of 27.8 kDa that contains a putative S-adenosyl-l-methionine (AdoMet) binding domain. It is also predicted to assume a seven-beta-strand fold which is indicative of DNA or small molecule methyltransferase activity^35,36^. The *N*-terminus of METTL7B is highly hydrophobic and serves to localize the protein to lipid droplets however, the physiological implications of this cellular localization are currently not known^37,38^. Additionally, METTL7B has been shown to play a role in the integrity of the Golgi apparatus^28^. To date, the function of human METTL7B remains a mystery despite several studies linking it to specific disease states, subcellular localizations, and cell processes.

In this report, we describe a number of experimental approaches that unequivocally demonstrate that METTL7B is an alkyl thiol methyltransferase. Using untargeted high-resolution proteomic analysis on partially purified rat liver microsomes (RLMs) possessing alkyl thiol methylation activity, we identified METTL7B as a strong candidate thiol methyltransferase. Gene modulation experiments in HepG2 and HeLa cells confirm that METTL7B expression is correlated with thiol methylation activity. Finally, purification of a His-GST-tagged recombinant protein and in vitro kinetic analysis established that METTL7B is indeed an alkyl thiol methyltransferase.

## Results

### Identification of METTL7B as a candidate thiol methyltransferase

Our interest in identifying TMT originated from our aspiration to identify the enzyme responsible for the methylation of the active thiol metabolite of clopidogrel^39^. We expanded on earlier research which attempted to purify TMT from rat liver microsomes (RLMs) using a series of chromatographic columns^22,23^. Investigatory non-targeted proteomic experiments were conducted to identify potential methyltransferase proteins in cation exchange resin elution fractions containing thiol methyltransferase activity. The major candidate protein, solely identified in active fractions, was rat METTL7B, a putative methyltransferase that is localized to the endoplasmic reticulum (ER) (Supplementary Data Table 1). Human METTL7B has a molecular weight of 27.8 kDa which is similar to the predicted TMT molecular weight of 28 kDa and shares 83% sequence homology with the rat ortholog, suggesting a potential conserved function^22,40^. Subsequently, we conducted several experiments, both in cell lines and in vitro*,* to support the initial findings.

### METTL7B gene expression modulation in HepG2 and HeLa cells and correlation with protein expression and methylation activity

**Figure 1:**
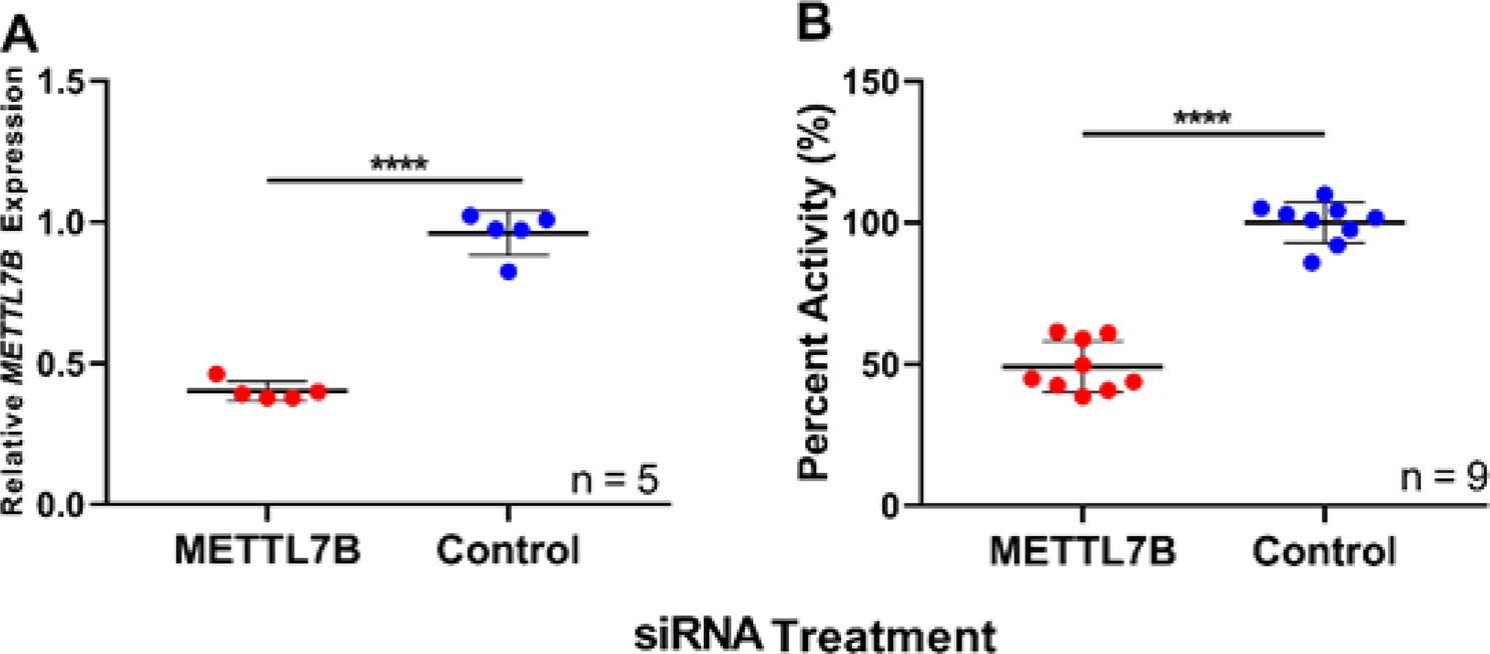
Knockdown of *METTL7B* gene expression in HepG2 cells: (**A**) RT-PCR data measuring *METTL7B* gene expression following treatment with *METTL7B* siRNA for 72 h compared to scramble treated control. (**B**) HepG2 *S-*methyl captopril formation following 24 h incubation in cells treated with *METTL7B* siRNA (72 h) compared to scramble control. All data is presented as the mean ± s.d. Individual data points are plotted from two (**A**) or three (**B**) separate experiments. Significance was determined using unpaired two-tailed *t* test. ****P < 0.0001.

Treating HepG2 cells with *METTL7B*-specific small interfering RNA (siRNA) decreased *METTL7B* mRNA expression by ~ 60% compared to cells treated with a scramble siRNA control (Fig. 1A). Incubation with the angiotensin converting enzyme inhibitor, captopril, a TMT probe substrate, following siRNA treatment, resulted, on average, in a 50% decrease in captopril methylation in HepG2 cells with reduced *METTL7B* gene expression compared to controls (Fig. 1B).

**Figure 2:**
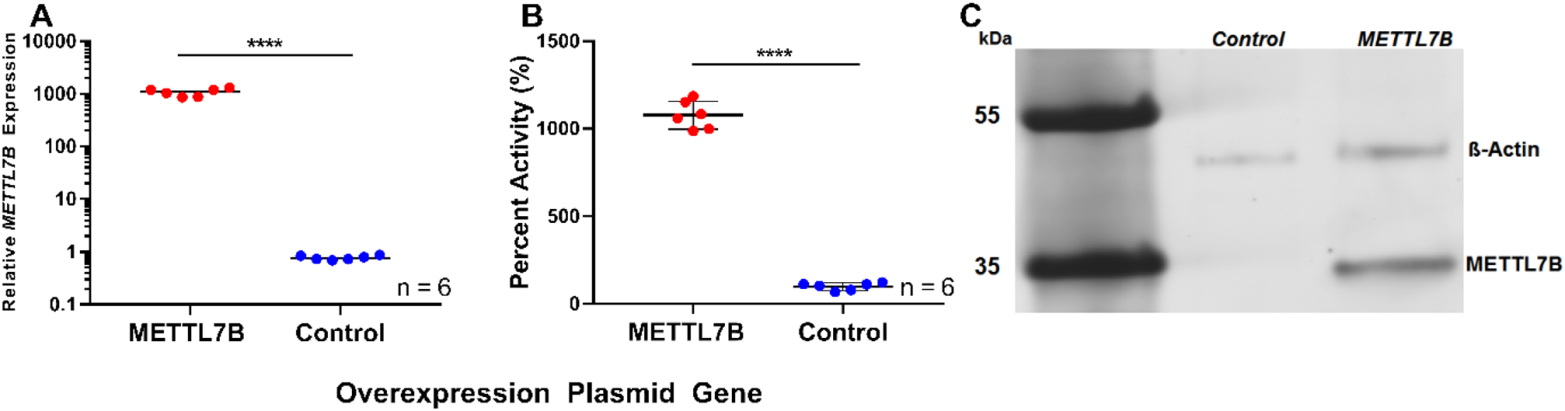
Overexpression of the *METTL7B* gene in HeLa cells: (**A**) RT-PCR data measuring *METTL7B* gene expression in HeLa cells treated with a *METTL7B* overexpression plasmid compared to control cells transfected with an empty expression vector. (**B**) *S*-methyl captopril measured 24 h following the addition of captopril (500 µM) to HeLa cells treated with *METTL7B* overexpression plasmid compared to control cells. (**C**) FLAG-tagged METTL7B expression is only observed in HeLa cells treated with the *METTL7B* overexpression plasmid and not in controls. Western blot using an anti-FLAG antibody confirmed the expression of METTL7B in HeLa cells. Full gel images are available in Supplementary Fig. 1. All data is presented as the mean ± s.d. Individual data points are plotted from two separate experiments. Significance was determined using unpaired two-tailed *t* test. ****P < 0.0001.

*METTL7B* mRNA expression increased over 1000-fold in HeLa cells treated with a constitutive overexpression plasmid containing the FLAG-tagged *METTL7B* gene sequence compared to cells treated with empty control plasmid as measured by RT-PCR (Fig. 2A). In addition, cells overexpressing METTL7B and treated with captopril produced tenfold higher *S*-methyl captopril compared to control cells (Fig. 2B). Anti-FLAG Western blot confirmed that cells transfected with the FLAG-tagged *METTL7B* plasmid overexpress METTL7B protein compared to control cells (Fig. 2C). These results reinforced the status of METTL7B as a potential alkyl thiol methyltransferase and supported the move forward with recombinant protein expression and characterization.

### Expression and purification of His-GST-METTL7B and His-GST-METTL7B-D98A

**Figure 3:**
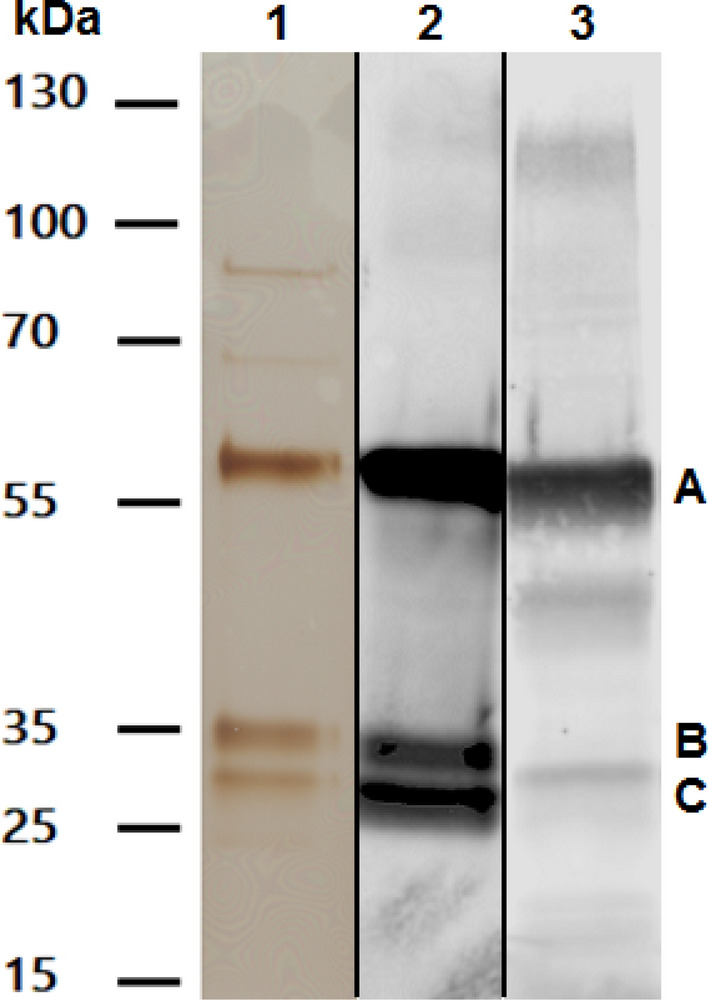
Purified His-GST-METTL7B: (Lane 1) SDS-PAGE silver stain of a representative gel showing purified His-GST-METTL7B (A). Gel lanes were loaded with a 1 µg total protein as determined by A_280._ (Lane 2) Anti-GST antibody western blot of purified His-GST-METTL7B (1 µg total protein). (Lane 3) Western blot using anti-METTL7B antibody of purified His-GST-METTL7B (A) using 0.1 µg total protein. Molecular weight markers are shown to the left. The lower molecular bands, marked by letters B and C, are fusion protein fragments containing the dual His-GST affinity tag.

The wild-type *METTL7B* gene sequence was inserted into a pET21 expression plasmid to express a unique fusion protein in *E. coli*. The recombinant protein, henceforth referred to as His-GST-METTL7B, has a molecular weight of ~ 57.5 kDa and contains a dual His-GST affinity/solubilization tag coupled to the *N*-terminus of the native METTL7B protein. The resulting purified protein fraction predominantly contains the His-GST-METTL7B fusion protein construct indicated in Fig. 3 by the letter “A”. His-GST-METTL7B was also identified by proteomic analysis in the protein fractions eluting from the GSH affinity column, and in the excised and digested 55 kDa band in lane 1 of Fig. 3 (Supplementary Data Tables 2 and 3). The lower bands around 30–35 kDa, indicated by “B” and “C” in Fig. 3, were identified by western blot as co-purified affinity tag GST-protein devoid of METTL7B. Full images of each individual gel are provided as Supplementary Figs. 2–4. Additionally, proteomic analysis demonstrated that the remaining background bands, particularly those above 55 kDa, are co-purified *E. coli* chaperone proteins (data not shown).

**Figure 4:**
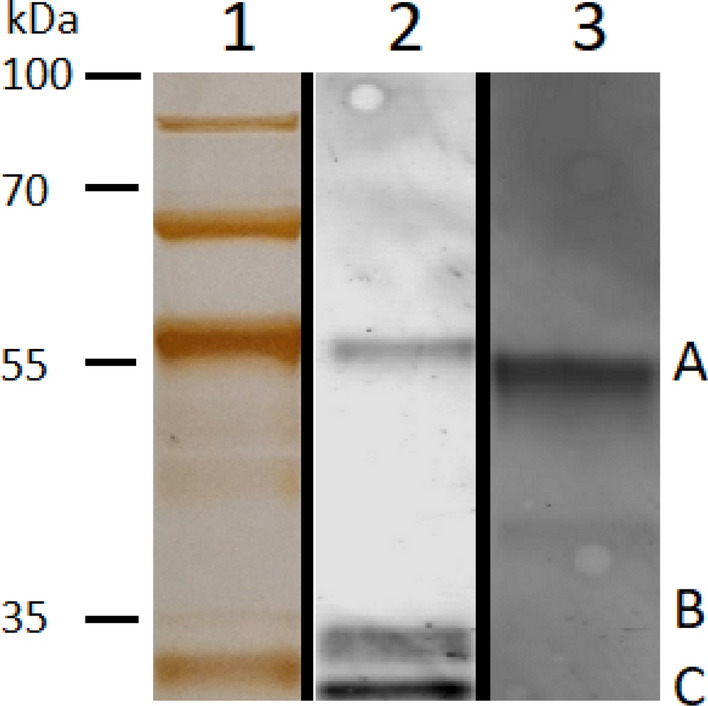
SDS-PAGE and western blot analysis of purified His-GST-METTL7B-D98A: (Lane 1) SDS-PAGE silver stain of a representative gel showing purified His-GST-METTL7B-D98A (A) (4.2 µg total protein) (Lane 2) Anti-GST antibody western blot of purified His-GST-METTL7B-D98A (3 µg total protein). (Lane 3) Anti-METTL7B antibody western blot of purified His-GST-METTL7B-D98A (4.3 µg total protein). Molecular weight markers are shown to the left. Upper bands, above 55 kDa, are co-purifying chaperone proteins as determined by proteomic analysis (data not shown). Lower molecular bands, marked by letters B and C, are fusion protein fragments containing the dual His-GST affinity tag.

A variant form of the His-GST-METTL7B fusion protein, incorporating a D98A single point mutation, was also expressed and purified. Incorporation of the mutation was confirmed by high-resolution proteomic analysis showing that Asp98 is indeed mutated to Ala98 (Supplementary Table 4). The mutant was created to potentially disrupt the binding of AdoMet to METTL7B and unequivocally prove that the methylation activity is attributed to METTL7B and not a highly active trace *E. coli* protein co-purified on the affinity columns. The mutant fusion protein, referred to as His-GST-METTL7B-D98A, displayed similar protein band patterns as the wild-type protein by SDS-PAGE and western blot analyses as demonstrated in Fig. 4. Full images of each gel are provided as Supplementary Figs. 5–7.

### Validation of His-GST-METTL7B thiol methyltransferase activity

**Figure 5:**
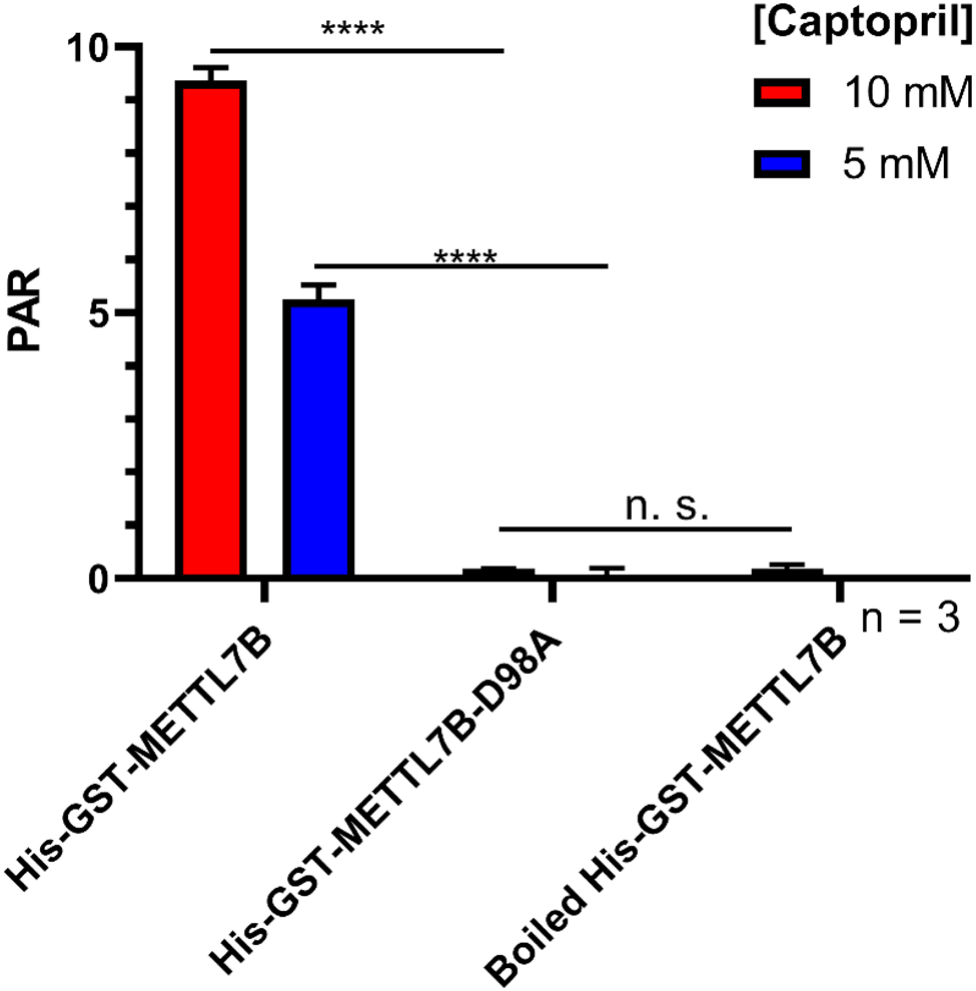
Thiol methyltransferase activity of purified His-GST-METTL7B and His-GST-METTL7B-D98A: Peak area of *S*-methyl captopril normalized to internal standard (PAR) at two substrate concentrations for both wild-type METTL7B (His-GST-METTL7B) and METTL7B containing a D98A point mutation (His-GST-METTL7B-D98A). Samples were protein-normalized to 0.15 mg/mL via A_280._ Data is presented as the mean ± s.d. Significance was determined using unpaired two-tailed *t* test. ****P < 0.0001.

Thiol methylation activity of His-GST-METTL7B-D98A was significantly compromised compared to the wild-type His-GST-METTL7B. As shown in Fig. 5, concentration-dependent formation of *S*-methyl captopril was observed upon incubation with purified wild-type His-GST-METTL7B but was absent upon incubation with purified His-GST-METTL7B-D98A. Methyltransferase activity was significantly improved upon pre-incubation of wild-type His-GST-METTL7B with dimyristoyl-*sn*-glycero-3-PG (DMPG) liposomes (1:85 w/w protein:liposome) while His-GST-METTL7B-D98A activity remained undetectable under similar incubation conditions as detailed in the methods.

The amount of *S*-methyl captopril formed in the presence of His-GST-METTL7B-D98A was not significantly different than background levels observed using boiled His-GST-METTL7B. This result confirmed that the observed methylation with the wild-type fusion protein is not due to a co-purified bacterial protein. Similar activity data was observed using *E. coli* homogenate from cells expressing His-GST-METTL7B or His-GST-METTL7B-D98A (Supplementary Figure 8) and containing any putative bacterial thiol methyltransferase enzymes. Since the mutant form was devoid of activity, all subsequent characterization experiments were conducted with the recombinant wild-type protein.

### Kinetic analysis of AdoMet and AdoHcy with His-GST-METTL7B

**Figure 6:**
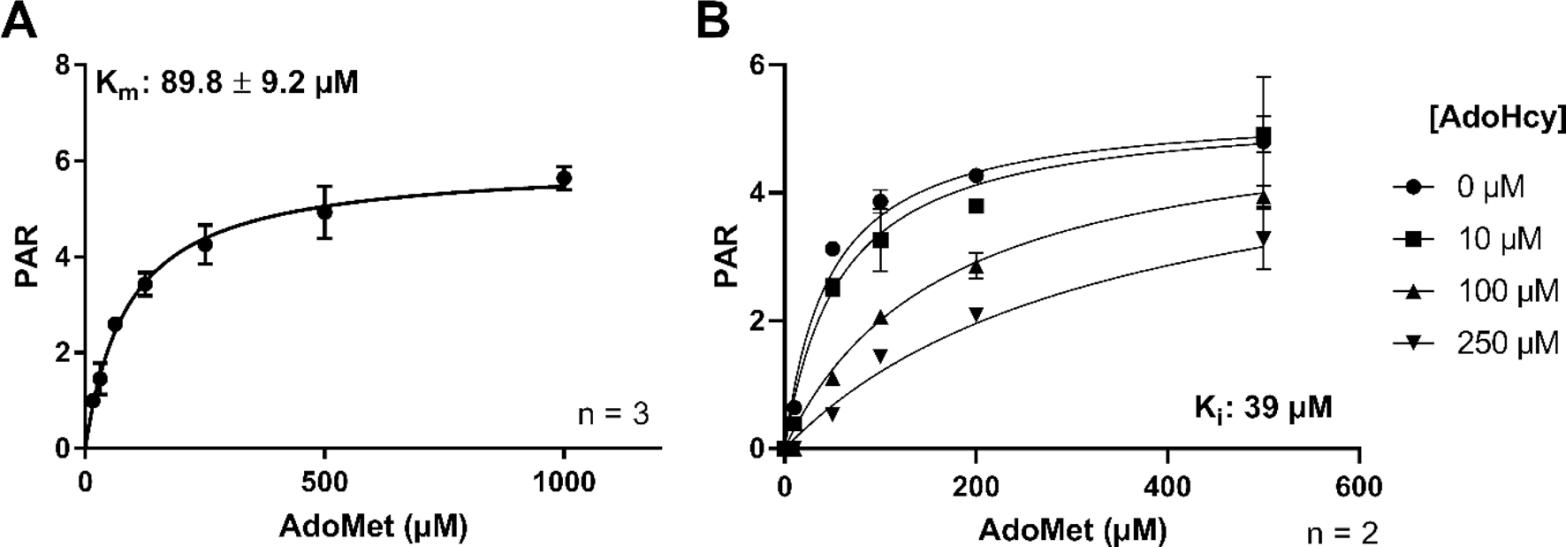
Captopril methylation by His-GST-METTL7B at various AdoMet concentrations in the presence or absence of AdoHcy: (**A**) His-GST-METTL7B catalyzed AdoMet-dependent methylation of captopril exhibits saturable Michaelis–Menten kinetics. Peak area ratio of methyl captopril to internal standard (PAR) versus concentration of AdoMet. (**B**) Inhibition of His-GST-METTL7B captopril methylation by AdoHcy. PAR versus AdoMet concentration curve at varying concentrations of AdoHcy. Data presented as mean ± s.d. n = 3 for (**A**) and average ± error (n = 2) for (B).

The Michaelis–Menten binding affinity parameter (K_m_) for AdoMet was measured via formation of *S-*methyl captopril as determined by LC–MS/MS. As demonstrated in Fig. 6, the AdoMet activity curve was saturable and exhibited classic Michaelis–Menten kinetics. The presence of AdoHcy inhibited methylation activity in a concentration-dependent manner and appeared competitive in nature as expected with AdoMet-dependent methyltransferases.

### His-GST-METTL7B substrate specificity

**Figure 7:**
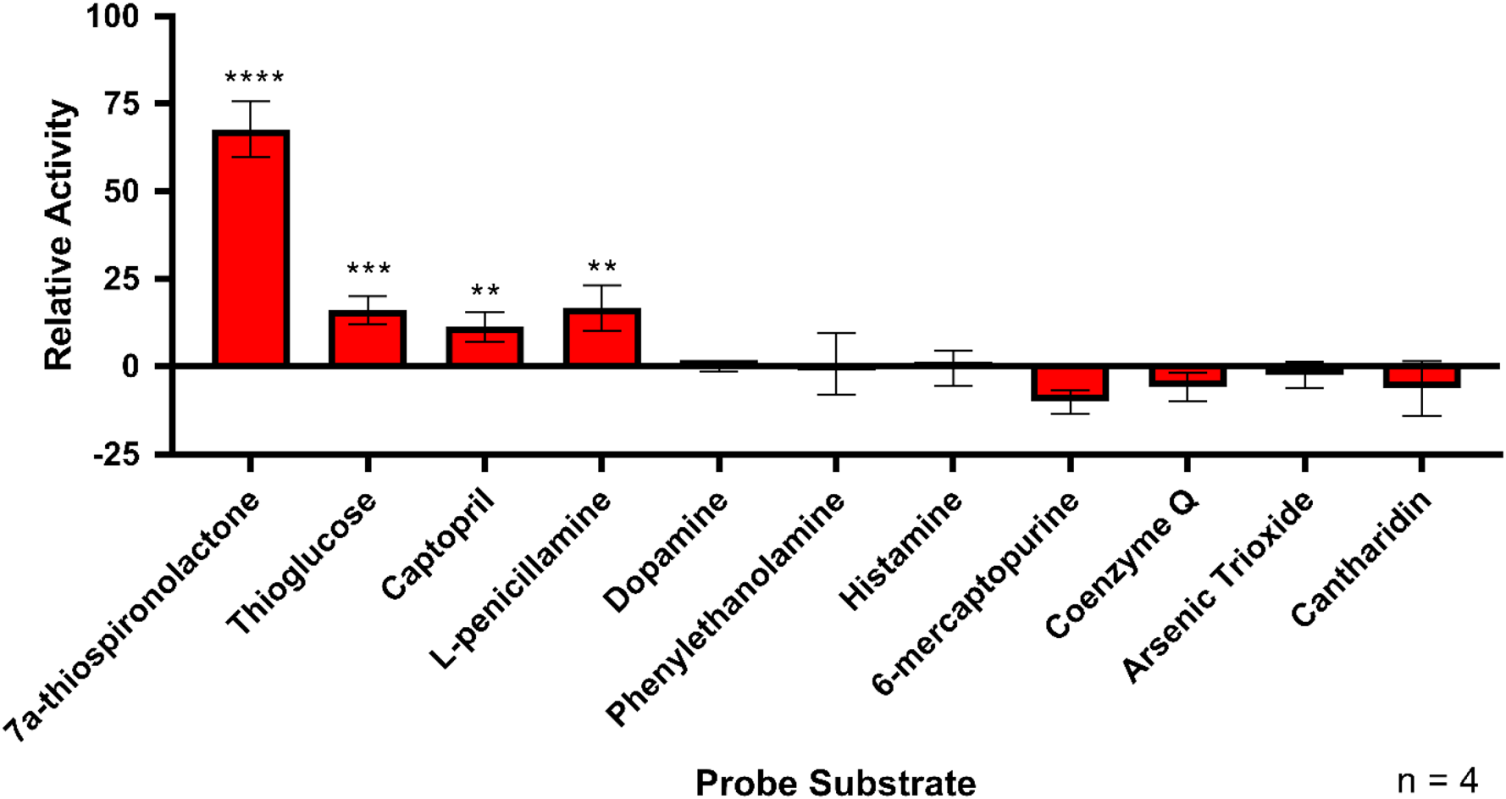
Semi-quantitative screening of select small molecule methyltransferase probe substrates. Multiple methyltransferase probe substrates were incubated for 1 h with His-GST-METTL7B. Formation of AdoHcy (indication of methyl transfer) was measured using the Promega MTaseGlo kit. Activity was normalized to dopamine, a catechol O-methyltransferase probe that showed no activity. All data is presented as the mean ± s.d. Significance was determined using unpaired two-tailed *t* test. ****P < 0.0001. ***P < 0.001. **P < 0.01.

Several known methyltransferase substrates and endogenous thiol compounds were screened for methyl metabolite formation with the Promega MTaseGlo kit using recombinant His-GST-METTL7B. Potential substrates were screened at concentrations at least three times higher than their reported K_m_ values to ensure detection of methylation activity. Non-enzymatic methylation, which has been observed for several potential substrates^41^, was accounted for by including boiled enzyme and buffer-only controls and subtracting that turnover from the activity observed with experimental samples. Qualitative screening results are presented in Table 1. A subset of the semi-quantitative screening results is presented shown in Fig. 7. Only aliphatic thiol compounds show significant methylation above baseline levels observed for dopamine, a negative control substrate.

**Table 1:** Relative methylating ability of His-GST-METTL7B with different probe substrates.

Substrates	Activity	Non-Substrates	Activity
7α-Thiospironolactone	+++	Dopamine	−
Dithiothreitol	+++	Phenylethanolamine	−
Thioglucose	++	Histamine	−
l-Penicillamine	++	6-mercaptopurine	−
d-Penicillamine	++	N-acetylcysteine	−
Hydrogen Sulfide	++	Arsenic Trioxide	−
Captopril	+	Cantharidin	−
Prasugrel Active Metabolite	+	Coenzyme M	−
		Coenzyme Q	−
		Cysteine	−
		Glutathione	−

### Kinetic analysis of His-GST-METTL7B

**Figure 8:**
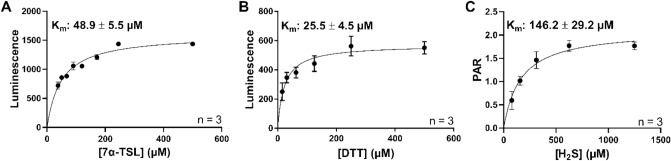
Rate of thiol methylation for His-GST-METTL7B with multiple substrates: (**A**) Luminescence versus concentration curves for 7α-thiospironolactone methylation as measured by AdoHcy formation. (**B**) Luminescence versus concentration curve for dithiothreitol methylation as measured by AdoHcy formation. (**C**) Peak area ratio (PAR) versus concentration curve for hydrogen sulfide methylation as measured by formation of methylsulfide. All data is presented as the mean ± s.d.

Kinetic parameters for a subset of the identified substrates were determined and are presented in Fig. 8. All substrates exhibited AdoMet-dependent and saturable methylation which was destroyed upon pre-boiling the enzyme. Kinetic parameters were measured under linear conditions with respect to incubation time and protein concentration (data not shown).

Methylation of hydrogen sulfide, and the resulting kinetic curve, were obtained using a mass spectrometric method directly measuring the formation of methylsulfide. 7α-thiospironolactone and dithiothreitol kinetic curves were obtained using the MTaseGlo kit (Promega) which measures the formation of AdoHcy, the byproduct of all AdoMet-dependent methylation reactions.

These substrates exhibited mid- to low-micromolar affinities to His-GST-METTL7B and classic Michaelis–Menten kinetics as evidenced by highly linear Eadie-Hofstee transformations of the data (Supplementary Data Figure 9).

## Discussion

The key finding of this study is that METTL7B specifically transfers a methyl group from AdoMet to small molecule alkyl thiol compounds, ranging in size from the gasotransmitter H_2_S to the steroid 7α-thiospironolactone. METTL7B emerged as a strong thiol methyltransferase candidate from proteomic analysis of partially purified fractions of rat liver microsomes displaying thiol methyltransferase activity. Subsequent bioinformatic analysis revealed that human METTL7B has high sequence identity with the rat isoform, a molecular weight of 27.8 kDa, and contains a putative AdoMet-binding domain. Silencing of *METTL7B* gene expression in HepG2 cells and overexpression in HeLa cells confirmed that gene and protein expression correlate with captopril methylation, a historically used probe substrate for TMT^6,15^. HepG2 and HeLa cells were ideal for gene knockdown and overexpression experiments respectively, because of their relatively high, and low, basal expression levels of *METTL7B* mRNA.

To biochemically characterize METTL7B and to prove that it possesses small molecule thiol methylation ability, we designed a unique plasmid to express and purify recombinant METTL7B fusion protein with two affinity tags (His-GST-METTL7B) which aid in solubilization and purification from an *E. coli* expression system. We also designed and purified a mutated form of the enzyme (His-GST-METTL7B-D98A) which includes a single point mutation altering a critical aspartate residue to an alanine. This mutation is proposed to negatively impact the binding affinity of AdoMet and should potentially result in a catalytically compromised enzyme^42^. Indeed, direct comparison of methyltransferase activity between purified wild-type His-GST-METTL7B and His-GST-METTL7B-D98A at similar protein concentrations and two different captopril concentrations proved that METTL7B accounts for the observed thiol methylation and not a highly active impurity present in the purified enzyme preparation.

It is worth noting that, during our attempts to solubilize METTL7B from *E. coli*, we discovered that 20% glycerol added to the solubilization buffer greatly stabilized METTL7B in solution. We also noted that the methylation activity was greatly enhanced by reconstituting His-GST-METTL7B in dimyristoyl-*sn*-glycero-3-PG (DMPG) liposomes prior to incubation with AdoMet and substrates as indicated in the methods section. DMPG liposomes were chosen due to their known beneficial effect on stabilizing lipid droplet-associated proteins^43^. Without glycerol and DMPG, His-GST-METTL7B was unstable and activity was variable and difficult to quantify, which perhaps contributed to the lack of characterization to date.

The wild-type His-GST-METTL7B fusion protein *S*-methylates multiple previously identified TMT-specific substrates in a time-, concentration-, and AdoMet-dependent manner. No activity was observed with a variety of known substrates for *N*-, *O*- and arsenic methyltransferases, as shown in Fig. 7. The compounds that undergo methylation conform to the substrate specificity and kinetic parameters previously determined for TMT using liver microsomes and erythrocyte membranes^6,15,16,18^. In general, METTL7B methylates compounds with a sterically unhindered aliphatic thiol functional group. Additionally, METTL7B does not methylate 6-mercaptopurine, a classic thiopurine methyltransferase (TPMT) substrate^44^. This further confirms that METTL7B catalyzes TMT-specific reactions rather than TPMT reactions. Consistent with prior reports, neither cysteine nor glutathione are substrates although, H_2_S is enzymatically methylated^20^. Methylation is also inhibited by the presence of AdoHcy, a hallmark of all AdoMet-dependent methyltransferases. It is important to note, that the His-GST-METTL7B fusion protein contains a glutathione *S*-transferase (GST) portion which may bind and sequester available glutathione, thus preventing methylation. To overcome this caveat, we used a high glutathione concentration to saturate GST binding sites and allow any potential methylation to occur. However, alternative non-GST fusion protein constructs containing METTL7B should be characterized for glutathione methylation and compared to His-GST-METTL7B in future work.

A potential key endogenous function of METTL7B is that it catalyzes the methylation of H_2_S to methylsulfide, a compound which has been detected in vivo^45^*,* but the exact function and activity of methyl sulfide are still unknown. While physiological concentrations of hydrogen sulfide are fiercely debated, the apparent hydrogen sulfide K_m_ measured for His-GST-METTL7B is similar to concentrations measured in vivo^46–48^. Currently, catabolism of hydrogen sulfide is believed to be primarily driven by oxidation^49^. However, this route of metabolism is potentially less prominent in organs outside of the gut and under hypoxic conditions, such as the interior of solid tumors^50,51^. Therefore, methylation of H_2_S by METTL7B may be a relevant route of metabolism in healthy tissue and more-so in specific disease states or tissue microenvironments. It is possible that *METTL7B* is upregulated to increase the rate of clearance of hydrogen sulfide, or perhaps to increase the formation of methylsulfide, in tumor cells. The effect of altered *METTL7B* expression in tumor cells is still unknown and further research is required to understand its role in the metabolism and homeostasis of hydrogen sulfide, especially in diseases that exhibit altered cellular redox states.

Finally, in addition to METTL7B, the human METTL7 family contains another paralog, methyltransferase-like protein 7A (METTL7A), an enzyme that also has an uncharacterized function. These two proteins share ~ 60% sequence identity which indicates a potential conserved function^40^. *METTL7A* and *METTL7B* have distinct gene expression profiles in different tissues and disease states. Therefore, while METTL7A may also possess thiol methyltransferase activity, among other activities, it is possible that each protein occupies a separate physiological role that requires more research.

## Conclusions

METTL7B possesses all the known characteristics of the elusive human alkyl thiol methyltransferase (TMT) and should be renamed as alkyl thiol methyltransferase. Human METTL7B clearly catalyzes the AdoMet-dependent methyl transfer to exogenous and select endogenous thiol compounds, distinct from TPMT and other small molecule methyltransferases. METTL7B is involved in the metabolism of H_2_S, which may be important in cancer and inflammation where gene expression is highly upregulated and H_2_S levels are altered. Future work will focus on elucidating the in vivo role METTL7B plays in healthy and diseased tissue.

## Methods

### Materials

Mammalian overexpression plasmids and siRNA were purchased from Origene (Rockville, MD). HepG2 and HeLa cells were obtained from ATCC (Manassas, VA) and maintained using accompanying protocols. Cell culture materials and lipofection reagents were purchased from ThermoFisher (Waltham, MA). Buffer salts were acquired from Sigma-Aldrich (St. Louis, MO) as well as methyltransferase probe substrates unless otherwise indicated. AdoMet and molecular biology kits, including the Q5 Site-Directed Mutagenesis Kit, were obtained from New England Biolabs (Ipswich, MA). Stellar competent cells were purchased from Takara (Mountain View, CA). LOBSTR-BL21(DE3) competent cells were bought from Kerafast (Boston, MA). CHAPS detergent and UPLC-grade solvents were obtained from Fisher Scientific (Hampton, NH). Sequencing grade porcine trypsin and MTaseGlo Methyltransferase Assays were purchased from Promega (Madison, WI). 1,2-Dimyristoyl-*sn*-glycero-3-PG (DMPG) was obtained from Cayman Chemical (Ann Arbor, MI). The active metabolite of prasugrel was a gift from Dr. Allan Rettie (University of Washington).

### Gene expression modulation

HepG2 cells were treated with Lipofectamine RNAiMax (Thermo Fisher Scientific, Waltham, MA) according to manufacturer protocols and optimized for transfection duration. Gene knockdown was achieved using a combination of the three separate small interfering RNA (siRNA) constructs present in the human *METTL7B* Trilencer-27 kit (Origene, Rockville, MD, Cat#: SR316261). *GAPDH* gene knockdown was used as a positive control and transfection with scramble siRNA acted as the negative control for all gene expression knockdown experiments. The scramble siRNA sense strand sequence is as follows: 5′ rCrGrUrUrArArUrCrGrCrGrUrArUrArArUrArCrGrCrGrUAT. All other siRNA sequences are proprietary to Origene.

Cells were transfected in 12-well plates using a reverse transfection protocol as previously published^52^. Briefly, *METTL7B* siRNA or non-targeted scramble siRNA was mixed with Lipofectamine RNAiMax in OptiMEM at room temperature for a final siRNA concentration of 50 nM. HepG2 cells were harvested using trypsin, pelleted, and resuspended to a final concentration of 200,000 cell/mL. Lipofectamine/siRNA stocks were added to culture plate wells, and further diluted by 1 mL of cells, for a final concentration of 10 nM siRNA. Cells were incubated in the transfection media for 72 h followed by RNA isolation or captopril methylation assays described below.

HeLa cells were treated with Lipofectamine 3000 (Thermo Fisher Scientific, Waltham, MA) according to the manufacturer protocol, optimized for transfection duration. Cells (2 × 10^5^ per well) were transfected in 12-well plates via reverse transfection where an overexpression plasmid (Origene, Rockville, MD, Cat#: RC203838) encoding a C-terminally FLAG-tagged *METTL7B* construct was mixed with P3000 reagent in OptiMEM at room temperature followed by Lipofectamine 3000. Transfection with an empty viral plasmid was utilized as a negative control^53^. Cells were incubated in transfection media for 48 h prior to RNA isolation or captopril methylation assays.

### Gene expression quantification

Total RNA was extracted using the MagMAX 96 Total RNA Isolation kit (Thermo Fisher Scientific, Waltham, MA) according to the manufacturer protocol. RNA quality (A_260_/A_280_) and concentration were determined using a NanoDrop spectrophotometer. Isolated RNA was used to create cDNA using the High-Capacity RNA-to-cDNA kit (Thermo Fisher Scientific, Waltham, MA) according to the manufacturer protocol. Subsequently, reverse-transcription polymerase chain reaction (RT-PCR) was conducted using an Applied Biosystems StepOnePlus Real-Time PCR System with proprietary TaqMan FAM reporter primers for *METTL7B, GAPDH,* and the housekeeping gene, *GusB*.

### S-Methyl captopril formation assay

After the appropriate transfection period for knockdown or overexpression described above, cells were washed with 1× PBS and treated with serum-free media containing 500 µM captopril. Cell media aliquots were sampled after 24 h and the S-methyl captopril metabolite was measured via liquid chromatography-tandem mass spectrometry (LC/MS–MS) and multiple reaction monitoring (MRM) after addition of internal standard (d_3_-*S*-methyl captopril).

The LC–MS/MS system used for captopril methylation analysis was a Waters Xevo TQS mass spectrometer paired with a Waters Acquity LC. Compound separation was achieved using a 2.1 × 100 mm Ascentis Express RP Amide column and 0.1% formic acid in water and 0.1% formic acid in methanol as solvents A and B respectively. Column temperature was maintained at 50 °C at all times. Chromatographic separation was obtained using the following gradient: solvent B held at 30% from 0 to 3 min, then increased to 90% from 3 to 7 min, followed by re-equilibration to the starting conditions for another 3 min for a total run time of 10 min. Flow rate was held constant at 0.2 mL/min.

*S*-Methyl captopril and the internal standard, d_3_-*S*-methyl captopril, were monitored in positive ionization mode. The monitored mass transitions m/z^+^ were 232.1 > 89 and 232.1 > 116 (*S*-methyl captopril) as well as 235.1 > 91.9 and 235.1 > 115.9 (internal standard). The MS conditions were as follows: collision energy 15 V, cone voltage 30 V, capillary voltage 3.2 kV, desolvation temperature 450 °C, desolvation gas flow 1000 L/h and cone gas 150 L/h.

### Wild-type and D98A mutant METTL7B expression and purification

Recombinant wild-type (His-GST-METTL7B) or mutant *METTL7B* (His-GST-METTL7B-D98A) were cloned in *E. coli* using a unique expression plasmid created in our lab. The expression plasmid backbone (pET21-10XHis-GST-HRV-dL5) was a gift from Marcel Bruchez (Addgene plasmid # 73214; http://n2t.net/addgene:73214; RRID:Addgene_73214). The human *METTL7B* gene sequence (Ensembl Transcript ENST00000394252.4) was inserted into the plasmid using BamHI and EcoRI restriction sites and general molecular biology techniques. The forward primer for insertion of the *METTL7B* sequence was 5′ CTAGCTAGGGATCCGCTCCGGCACCGGCTCCGGCACCGGCACCGATGGATATTTTAGTGCCATTGTTACAGCTT 3′. The reverse primer for insertion of the *METTL7B* sequences was 5′ CTAGCTAGGAATTCTTATTAACGCGTCTTGACGGCTTT 3’.

The Q5 Site-Directed Mutagenesis Kit (New England Biolabs, Ipswich, MA, Cat# E0554S) was used to introduce a missense mutation in the existing pET21 expression plasmid encoding for wild-type His-GST-METTL7B. Custom forward (5′ TACGTGCCTT**GcC**CCTAATCCGCATTTTGAAAAATTCTTGAC 3′) and reverse (5′ ACCCGGCAGCCCGGAGGA 3′) non-overlapping mutagenic primers were designed with New England Biolab's online primer design tool, NEBaseChanger. By non-homologous base-pairing the mutagenic forward primer incorporates a single A-to-C nucleotide substitution, as indicated by the lowercase type-set in the bolded codon encoding the D98 position, during PCR amplification.

The wild-type His-GST-METTL7B pET21 expression plasmid was combined with the mutagenic primers and necessary kit additives. The reaction subsequently underwent PCR amplification using an Applied Biosystems 2720 Thermal Cycler and an annealing temperature of 71 °C. Immediately after PCR amplification, the template primer was degraded, and the amplified mutant expression plasmid was ligated using a one-pot protocol according to the manufacturer’s protocols.

All plasmid inserts were validated by sequencing through Eurofins Genomics (Louisville, KY) and sequencing histograms were analyzed using FinchTV software. The forward sequencing primer was 5′ GGGCTGGCAAGCCACGTTTGGTG 3′ and the reverse sequencing primer was 5′ ATGAATGAACACCTTCACCATGC 3′. A second set of sequencing primers was used to confirm the correct incorporation of the D98A point mutation. The second set forward sequencing primer was 5′ AATCCAGCAAGTATATAGCATGGC 3′ and the reverse sequencing primer was 5′ ACAAGGGTGCATACAACAAC 3′. The final expression plasmids place a N*-*terminal His-GST affinity tag onto the wild-type, or mutant, METTL7B protein sequence separated by a 10-residue poly-alanine-proline (AP)_5_ linker.

Expression plasmids were propagated using heat-shocked Stellar cells (Takara, Mountain View, CA). Plasmids validated by sequencing were used to transform competent LOBSTR-BL21(DE3) *E. coli* (Kerafast, Winston-Salem, NC) via heat shock. Unless otherwise noted, *E. coli* cells were cultured in an orbital shaker at 250 rpm, 37 °C, and in the presence of 100 µg/mL ampicillin.

To express recombinant protein, LOBSTR-BL21(DE3) overnight cultures were added to ampicillin-containing TB expression media at a ratio of 1:100. Briefly, cells were grown for 3 h under normal growth conditions. Then, METTL7B production was induced via addition of isopropyl ß-D-1-thiogalactopyranoside (IPTG) to a final concentration of 1 mM. The temperature was reduced to 15 °C and the cells were grown for an additional 24 h. Cells were harvested via gentle centrifugation and the resulting pellets were stored at − 80 °C until future processing.

Frozen cell pellets were thawed on ice in a 4 °C cold cabinet overnight prior to resuspension in lysis buffer (50 mM KPi pH 7.0, 20% glycerol, 150 mM NaCl, 10 mM CHAPS, EDTA-free Halt Protease Inhibitor Cocktail) supplemented with 100 µg/mL lysozyme (Sigma Aldrich, St. Louis, MO). The lysate was rotated end-over-end at 4 °C until it became extremely viscous followed with the addition of 100 µg/mL DNA Nuclease I (Sigma Aldrich, St. Louis, MO) and rotated at 4 °C or until no longer viscous. The lysate was then centrifuged at 48,000 g for 30 min at 4 °C and the resulting supernatant was retained for subsequent purification steps.

Purification was conducted using the ӒKTA start chromatography system (GE Healthcare, Chicago, IL). Cell lysate supernatant was applied to a pre-packed and conditioned HisPur Ni–NTA column (ThermoFisher, Waltham, MA) overnight at a low flow rate (0.5 mL/min). The column was subsequently washed with Ni–NTA purification buffer (50 mM KPi pH 7.0, 20% glycerol, 10 mM CHAPS, 300 mM NaCl) containing 50 mM imidazole until A_280_ readings stabilize. Protein was eluted from the column with purification buffer containing 300 mM imidazole until A_280_ readings stabilized.

The HisPur Ni–NTA column eluent was directly applied to a pre-conditioned GSTrapFF column at a flow rate of 1 mL/min for 4 h. The column was then washed with GSTrapFF purification buffer (50 mM KPi pH 7.0, 20% glycerol, 10 mM CHAPS, 150 mM NaCl) until A_280_ had decreased to baseline. Recombinant protein was eluted from the column using purification buffer containing 10 mM reduced glutathione and adjusted to pH 8.0. Pooled eluents were concentrated using Amicon Centriprep 10 K molecular weight cutoff centrifugal filter units. Final protein concentration was determined by A_280_ measurement and stocks were aliquoted and stored at − 80 °C until future use.

### In vitro captopril methylation using recombinant METTL7B

In vitro captopril methylation was conducted using purified wild-type or mutant METTL7B fusion protein. The reaction buffer (50 mM KPi pH 7.0, 10 mM CHAPS, 20% glycerol, 150 mM NaCl, and 9 mg/mL DMPG) was placed in a sonication water bath until the solution was clear to help form DMPG liposomes. Recombinant enzyme was reconstituted with the reaction buffer at an optimized ratio of 85:1 DMPG:METTL7B (w/w) and allowed to incubate on ice for 30 min. Following the addition of captopril, the enzyme was pre-equilibrated at 37 °C for 2 min before the addition of AdoMet to a reaction volume of 150 µL. The final concentration of captopril was 10 mM and the final concentrations of AdoMet were 15.625, 31.25, 62.5, 125, 250, and 1000 µM. The reaction was incubated for 25 min and quenched via addition of 15% (w/v) zinc sulfate in a 1:5 ratio. The quenched solution was incubated on ice for 10 min followed by a 1:6 addition of a saturated barium hydroxide solution containing the d_3_-*S*-methyl captopril internal standard. Following a second 10-min incubation on ice, the solution was centrifuged at 5000 g for 15 min to pellet all precipitated proteins and salts. The supernatant (75 µL) was transferred to an opaque polypropylene strip-well tube containing 5 µL of 2 M sodium hydroxide. Unreacted captopril was derivatized at room temperature for 1 h in the dark via addition of 20 µL of 2.5 M maleimide to reduce ion suppression from captopril. Derivatized samples were centrifuged and the supernatant was analyzed by LC–MS/MS as described above.

### In vitro AdoHcy inhibition of captopril methylation using recombinant METTL7B

Protein concentration (wild-type or mutant) and reconstitution with DMPG liposomes were identical to the captopril methylation assay above. The final concentration of captopril was maintained at 10 mM and the final concentrations of AdoMet were 10, 50, 100, 200, or 500 µM. At each AdoMet concentration, AdoHcy was added at concentrations of 0, 10, 100, or 250 µM. Reaction volumes, conditions, sample processing, and LC–MS/MS analysis were conducted as previously for the captopril assay.

### In vitro hydrogen sulfide methylation using recombinant His-GST-METTL7B

Protein concentration and incubation with DMPG liposomes were conducted as described above for captopril methylation. All steps were conducted in a glove box under an atmosphere of nitrogen unless otherwise indicated. Recombinant enzyme was aliquoted into a polypropylene deep-well plate on ice along with AdoMet and sodium hydrosulfide (NaSH) for a final volume of 150 µL and 0.09 mg/mL and 83.3 µM for protein and AdoMet concentrations respectively. The plate was capped with a silicon mat and placed in a 37 °C water bath for 45 min under normal atmosphere. After incubation, the plate was placed back on ice under nitrogen in the glovebox and quenched via a 1:15 addition of 0.3 M sodium hydroxide. 110 µL of the quenched reaction solution was added to 50 µL of 20 mM monobromobimane (MBB), based off of published H_2_S derivatization method^54–56^. Once capped under nitrogen, the reaction plate was incubated at room temperature on an orbital shaker at 450 rpm for 30 min.

The MBB derivatization reaction was quenched by addition of 50 µL of 200 mM 5-sulfosalicylic acid containing 10 µL of the ethyl 2-aminothiazole carboxylate (EATC) as the internal standard. Protein was precipitated by addition of 15% (w/v) zinc sulfate and barium hydroxide as detailed above. Samples were centrifuged at 4000*g* for 15 min and the supernatant was analyzed by LC–MS/MS.

The LC–MS/MS system used for hydrogen sulfide methylation analysis was a Waters Xevo TQS mass spectrometer paired with a Waters Acquity LC. Compound separation was achieved using a 2.1 × 150 mm Acquity UPLC BEH Shield RP column and 0.2% acetic acid in water and 0.2% acetic acid in acetonitrile as solvents A and B respectively. Column temperature was maintained at 25 °C at all times. Chromatographic separation was obtained using the following gradient: solvent B was held at 40% from 0 to 1 min, ramped to 90% from 1 to 3.5 min, held at 90% from 4.5 to 5 min followed by re-equilibration to the starting conditions for another minute. Flow rate was held constant at 0.3 mL/min.

Derivatized methylsulfide and the internal standard, EATC, were monitored in positive ionization mode. The monitored mass transitions m/z+ were 239.22 > 175.24 and 239.22 > 192.2 (derivatized methylsulfide) as well as 173.17 > 72.11 and 173.17 > 127.06 (internal standard). The MS conditions were as follows: collision energy 24, 10, 24, 16 V for each transition respectively, cone voltage 56 V, capillary voltage 2.9 kV, desolvation temperature 450 °C, desolvation gas flow 1000 L/h and cone gas 150 L/hr.

### Protein purity analysis

All SDS-PAGE silver stain analysis was conducted using NuPAGE 4–12% Bis–Tris gels in the XCell SureLock Mini-Cell Electrophoresis system using PageRuler Plus Prestained Protein Ladder as a molecular weight marker. Gels were performed at room temperature, at a constant 200 V, and developed using previously published silver staining protocols^57^.

All western blot analyses were conducted using the XCell SureLock Mini-Cell Electrophoresis system, PageRuler Plus Prestained Protein Ladder, and NuPAGE 10–20% Tricine gels. A primary antibody incubation was conducted overnight using the suggested dilution factors (1:500 or 1:1,000) for the rabbit anti-METTL7B (Invitrogen, Carlsbad, CA, Ref: PA5-58478), anti-FLAG (Cell Signaling, Danvers, MA, #14793), anti-GST (Cell Signaling, Danvers, MA, #2625), or anti-ß actin (Cell Signaling, Danvers, MA, #4970) antibodies. The secondary antibody incubation was conducted for 1 h at room temperature using IRDye 680RD goat anti-rabbit antibody (LiCor, Lincoln, NE, P/N: 926-68071). Western blots were scanned using an Odyssey gel scanner. Blot images were visualized using Image Studio Version 4.0 software.

### Tryptic digest

In-gel tryptic digests of silver stained SDS-PAGE gels were conducted following the method published by Shevchenko^57^. Briefly, the protein band was excised from the gel and dehydrated with neat acetonitrile. Protein bands were then treated with 10 mM dithiothreitol (DTT) solution and incubated at 56 °C to reduce all proteins. The reduced bands were treated with 55 mM iodoacetamide at room temperature in the dark to alkylate all exposed cysteine side chains. Finally, the bands were incubated overnight at 37 °C with 13 ng/µL trypsin-containing solution. Digested peptides were extracted from the gel bands the following day and concentrated in a centrifugal evaporator. Concentrated peptides were analyzed using a Finnigan LTQ Orbitrap coupled to a Waters Acquity LC. Peptides were separated using a 1 × 150 mm Acquity UPLC CSH C18 column and 0.1% formic acid in water and 0.1% formic acid in acetonitrile as solvents A and B. Separation was achieved using the following gradient: solvent B was held at 5% for the first two minutes, increased to 40% over the next 90 min, increased to 90% over the next five minutes and held for an additional 8 min, then re-equilibrated over five minutes. The flow rate was held at 0.06 mL/min and flow as diverted to the mass spectrometer from 2 to 95 min.

Peptides were analyzed using a data dependent scan method in positive mode. The initial high-resolution scan from 300 to 2000 m/z was conducted in the FTMS with 60,000 resolution. Four dependent scans were completed in the ion trap to obtain fragmentation. Dynamic exclusion was enabled which excluded the top 25 most intense ions after they had been selected twice over a four second window. The following mass spectrometer settings were used: sheath gas flow rate was 12 arb, spray voltage was 3.5 kV, capillary temperature was 350 °C, capillary voltage was 22 V, and tube lens voltage was 100 V. Peptides observed in the tryptic digest were identified via ProteinProspector.

### Substrate screening

Substrate screening was primarily conducted using the MTaseGlo Assay (Promega, Madison, WI). Briefly, recombinant METTL7B was reconstituted with DMPG as described above for the captopril assay. AdoMet was added to the METTL7B protein stock for a final concentration of 50 µM and aliquoted into a 384-well plate. Substrate was added to each well and the plate was covered using Parafilm before incubating at 37 °C for 1 h. The final concentration of all substrates was 1 mM except for histamine (35 µM) and arsenic trioxide (50 µM). The incubation was quenched by a 1:5 addition of 0.5% trifluoro acetic acid. Following quenching, wells were processed according to the manufacturer’s protocol and luminescence was recorded for each well using a Synergy HTX Multi-Mode Reader (BioTek, Winooski, VT). All potential substrates were incubated with activity buffer in the absence of enzyme, boiled His-GST-METTL7B, or active His-GST-METTL7B. Luminescence due to non-enzymatic methylation observed in the buffer-only control was subtracted from luminescence observed in the presence of active enzyme. An identical method was used to measure enzyme kinetics for dithiothreitol and 7α-thiospironolactone, except varying substrate concentrations. The final concentrations used for dithiothreitol kinetic analysis were 15.625, 31.25, 62.5, 125, 250, and 500 µM. The final concentrations used for 7α-thiospironolactone kinetic analysis were 38, 50.6, 67.5, 90, 120, 171.5, 245, and 500 µM.

### Data analysis

Changes in mRNA expression levels following siRNA or plasmid treatment were determined using the ΔΔC_T_ method^58^. In this method, *METTL7B* cycle threshold (C_t_) values are normalized to *GusB* C_t_ values in all samples, yielding a ΔC_T_ value. Relative gene expression changes are then calculated between treated and control cells using 2^−ΔΔCT^. All experiments were conducted with biological triplicates and repeated at least two times on two different days. All data is reported as the mean ± standard deviation, however individual data points from multiple experiments are presented when appropriate. Statistical significance was determined by a two-tailed unpaired *t*-test with a threshold *P* value of 0.05. Kinetic parameter K_m_ and K_i_ (for AdoHcy) values were obtained by non-linear regression analysis using GraphPad Prism, version 8.3.1 for Windows (GraphPad Software, La Jolla, CA).

## Supplementary Information

Supplementary Information

## Data Availability

The proteomic data that support the findings of this study are available from PeptideAtlas, tagged as "pET21 METTL7B" and with a dataset identifier of "PASS01534". All other data are available from the corresponding author upon request. Unique materials used when conducting the experiments detailed in this study are available from the corresponding author upon reasonable request.
