# Supplementary material for: Human METTL7B is an alkyl thiol methyltransferase that metabolizes hydrogen sulfide and captopril
Source: Sci Rep. 2021 Mar 1;11:4857. doi: 10.1038/s41598-021-84218-5 (PMC7921093; doi:10.1038/s41598-021-84218-5)
Supplement: Supplementary file 1 — Supplementary Information [file 41598_2021_84218_MOESM1_ESM.docx]

**Supplementary Data for** *“*Human METTL7B is an alkyl thiol methyltransferase that metabolizes hydrogen sulfide and captopril”

Benjamin J. Maldonato, Drake A. Russell, and Rheem A. Totah*

University of Washington

Department of Medicinal Chemistry

1959 NE Pacific Ave,

Box 357610

Seattle, WA 98195

Corresponding Author: Dr. Rheem A. Totah

Email: [rtotah@uw.edu](mailto:rtotah@uw.edu)

Phone: 206-543-9481

**Supplementary Table 1: Proteins Identified in TMT-active in Rat liver Microsomal Purification Fraction**

**
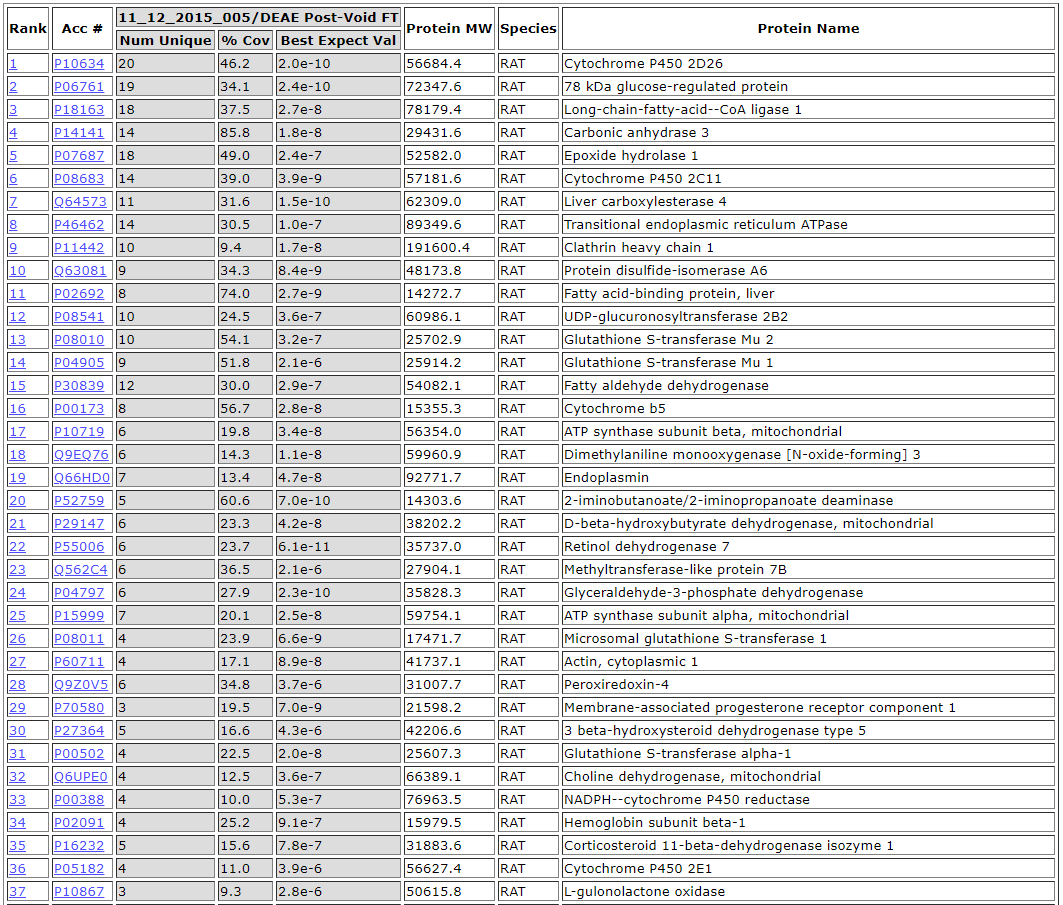
**

**
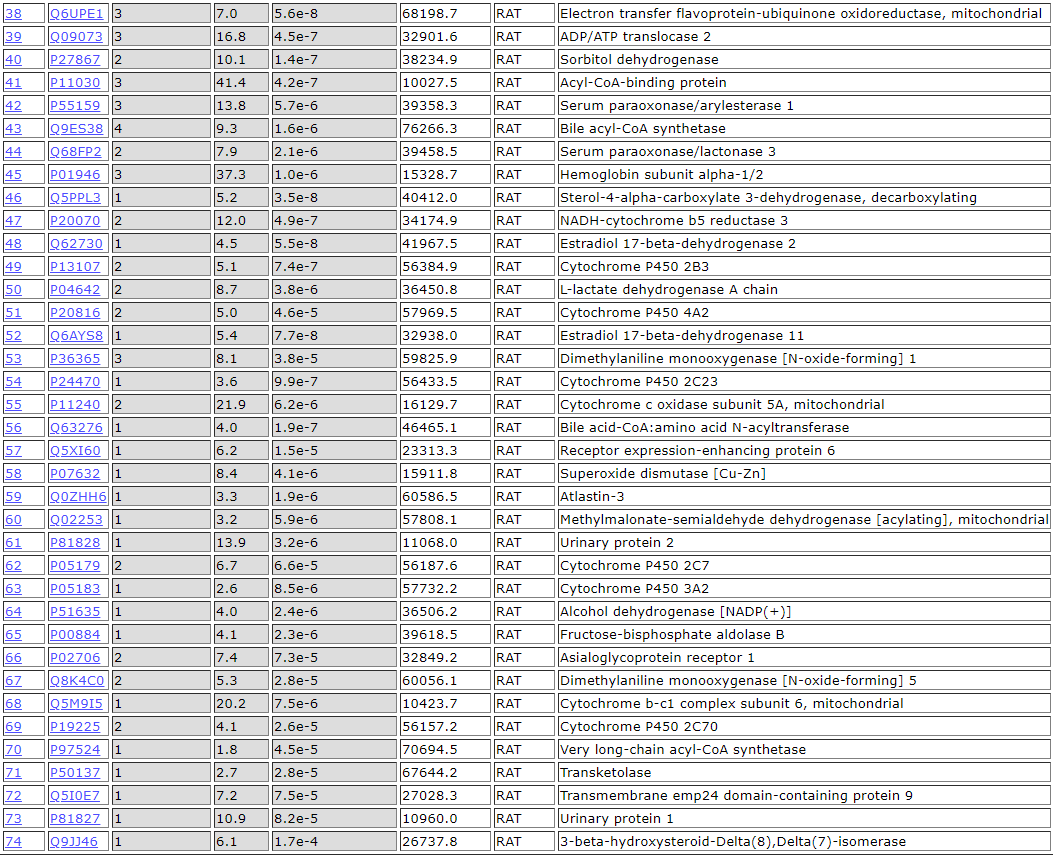
**

**Supplementary Table 2: Human Peptides Identified from Purified His-GST-METTL7B**

**
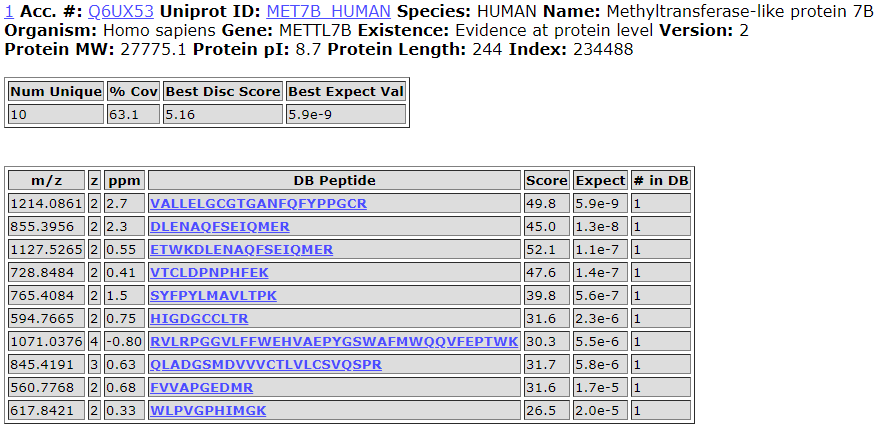
**

**Supplementary Table 3: METTL7B Peptides Identified from SDS-PAGE In-Gel Digest**

**
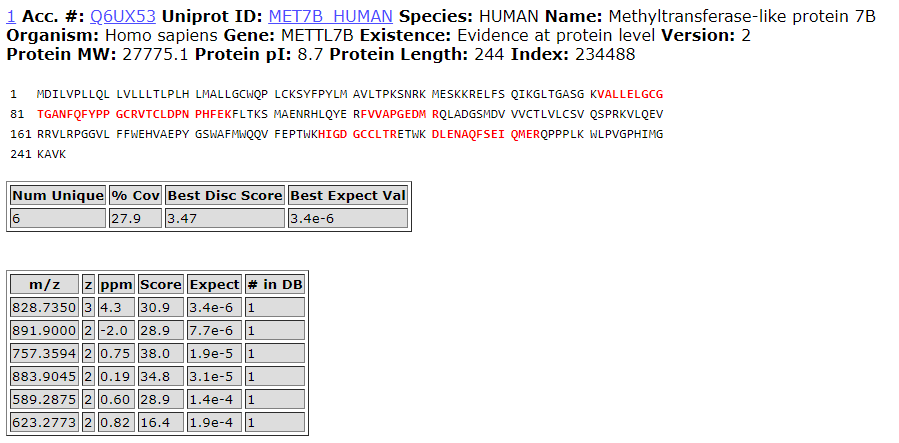
**

**
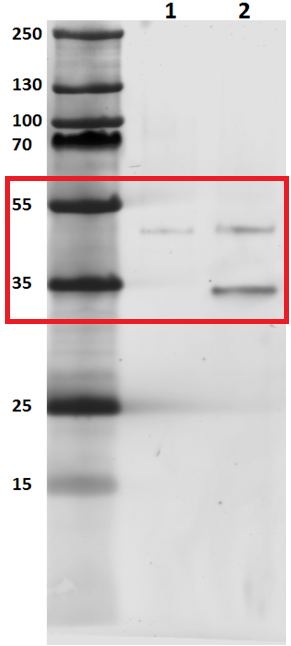
**

**Supplementary Figure 1. Western blot analysis of HeLa cell lysate:** Full image of anti-FLAG and anti-ß-actin western blot analysis of HeLa cells treated with either an empty control plasmid (Lane 1) or a *METTL7B* overexpression plasmid (Lane 2). FLAG-tagged METTL7B is around 31 kDa while ß-actin is around 42 kDa. Equivalent amounts of protein were loaded per lane as determined by BCA. The portion of the gel presented in the main manuscript is boxed in red.


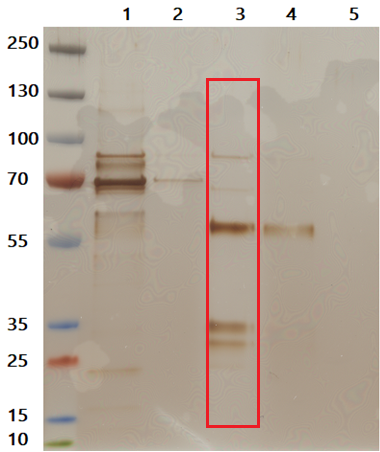


**Supplementary Figure 2. SDS-PAGE silver stain analysis of His-GST-METTL7B purification:** Full image of an SDS-PAGE analysis of the GSTrapFF purification of His-GST-METTL7B expressed and isolated from LOBSTR *E. coli.* The lane contents are as follows: Lane 1) HisPur Ni-NTA resin eluent following GSTrapFF loading. Lane 2) Pooled GSTrapFF resin wash fractions. Lane 3) Pooled GSTrapFF resin eluent fractions. Lane 4) GSTrapFF 6 M guanidine HCl wash. Lane 5) GSTrapFF 1% (v/v) Triton X-100 wash. Each lane was loaded with a total of 1 µg total protein as determined by A_280._ Lane 3 in the present gel was included as lane 1 in Figure 3 in the main manuscript. The portion of the gel presented in the main manuscript is boxed in red.


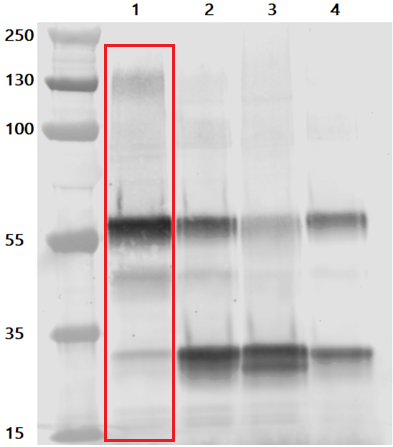


**Supplementary Figure 3. Anti-METTL7B western blot analysis of His-GST-METTL7B cleavage:** Full image of anti-METTL7B primary antibody western blot analysis of HRV3C-mediated cleavage of His-GST-METTL7B. The lane contents are as follows: Lane 1) Non-cleaved His-GST-METTL7B. Lane 2) HRV3C-treated His-GST-METTL7B. Lane 3) Flowthrough of GSTrapFF exposed to HRV3C-treated His-GST-METTL7B. Lane 4) Eluent of GSTrapFF exposed to HRV3C-treated His-GST-METTL7B. Each lane was loaded with a total of 1 µg total protein as determined by A_280._ Lane 1 in the present gel was included as lane 3 in Figure 3 in the main manuscript. The portion of the gel presented in the main manuscript is boxed in red.


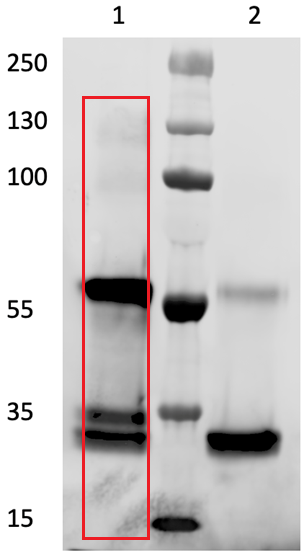


**Supplementary Figure 4. Anti-GST western blot analysis of His-GST-METTL7B cleavage:** Full image of anti-GST primary antibody western blot analysis of HRV3C-mediated cleavage of His-GST-METTL7B. Molecular weight markers are shown in the middle lane. Lane 1 contains 1 µg of non-cleaved His-GST-METTL7B. Lane 2 contains 1 µg of His-GST-METTL7B exposed to HRV3C protease. Lane 1 in the present gel was included as lane 2 in Figure 3 of the main manuscript. The portion of the gel presented in the main manuscript is boxed in red.

**
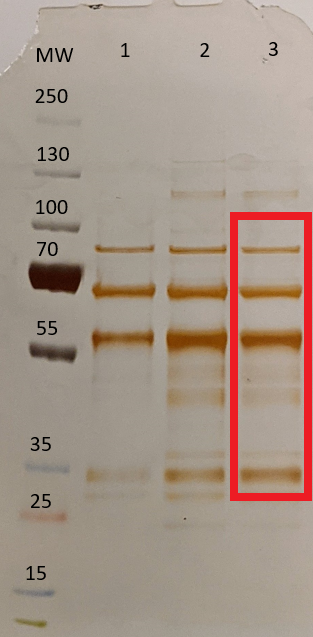
**

**Supplementary Figure 5. SDS-PAGE silver stain analysis of purified His-GST-METTL7B and purified His-GST-METTL7B-D98A:** Full image of SDS-PAGE analysis of purified aliquots of His-GST-METTL7B and His-GST-METTL7B-D98A. Molecular weight markers (PageRuler Plus) are shown in the lanes labeled “MW” and molecular weights are noted. Each lane was loaded with 4.2 µg of protein as determined by A_280_. Lane 1 contains purified His-GST-METTL7B and lanes 2 and 3 contain separate repeat purifications of His-GST-METTL7B-D98A. Lane 3 in the present gel was included as lane 1 in Figure 4 of the main manuscript. The portion of the gel presented in the main manuscript is boxed in red.

**
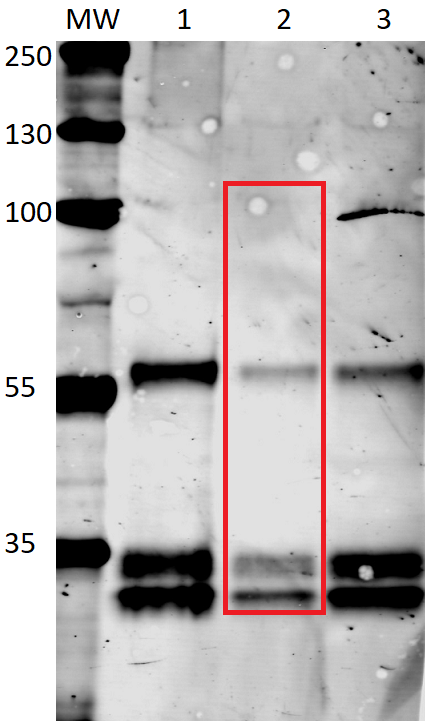
**

**Supplementary Figure 6. Anti-GST western blot analysis of His-GST-METTL7B-D98A:** Full image of anti-GST primary antibody western blot analysis of His-GST-METTL7B-D98A is shown in lanes 2 and 3. Molecular weight markers (PageRuler Plus) are shown in the lanes labeled “MW” and molecular weights are noted. Lane 1 contains 5 µg of purified His-GST-METTL7B. Lanes 2 and 3 contain 3 µg and 4.3 µg of purified His-GST-METTL7B-D98A, respectively. Lane 2 of the present gel is included in Figure 4 of the main manuscript. The portion of the gel presented in the main manuscript is boxed in red.

**
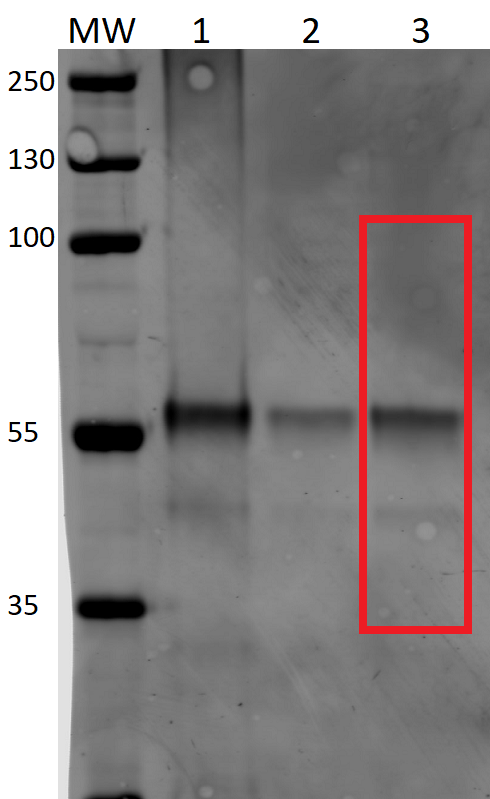
**

**Supplementary Figure 7. Anti-METTL7B western blot analysis of His-GST-METTL7B-D98A:** Full image of anti-METTL7B primary antibody western blot analysis of His-GST-METTL7B-D98A is shown in lanes 2 and 3. Molecular weight markers (PageRuler Plus) are shown in the lanes labeled “MW” and molecular weights are noted. Lane 1 contains 5 µg of purified His-GST-METTL7B. Lanes 2 and 3 contain 3 µg and 4.3 µg of purified His-GST-METTL7B-D98A, respectively. Lane 3 of the present gel is included in Figure 4 of the main manuscript. The portion of the gel presented in the main manuscript is boxed in red.

**Supplementary Table 4: Human Peptides Identified from Purified His-GST-METTL7B-D98A
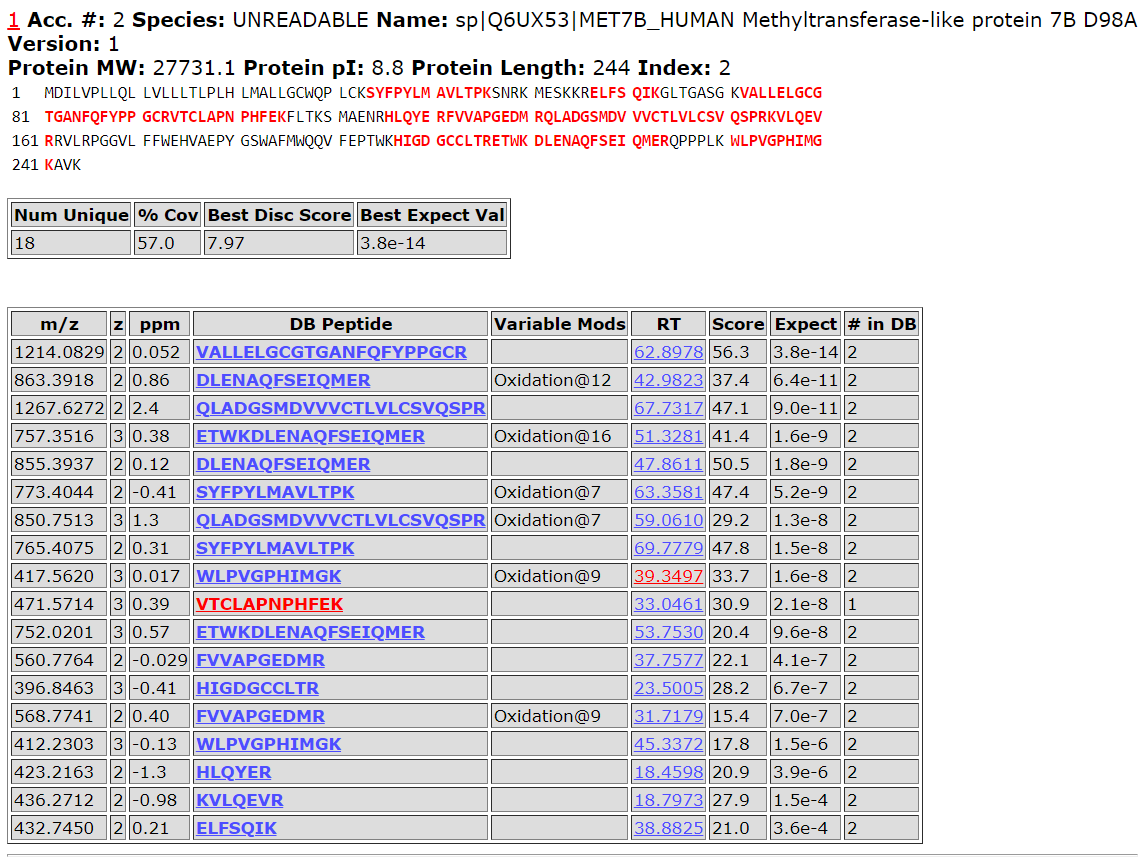
**

**
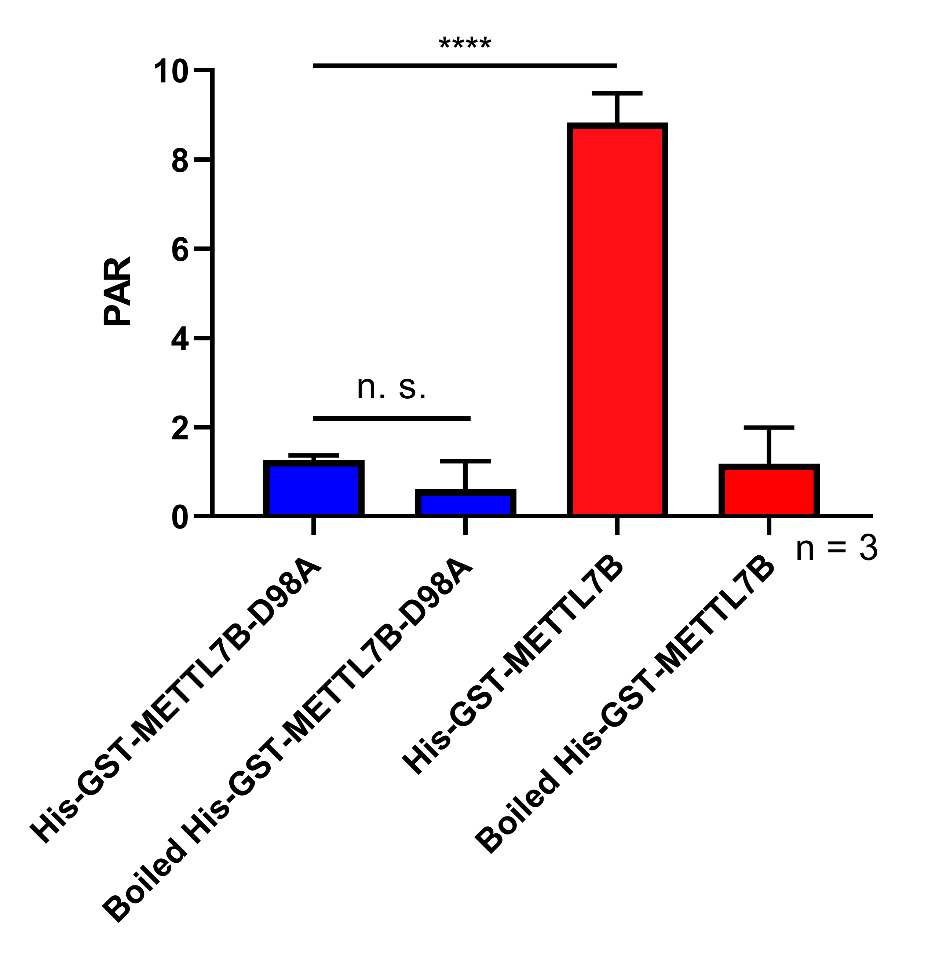
**

**Supplementary Figure 8. Thiol methyltransferase activity of His-GST-METTL7B and His-GST-METTL7B-D98A in induced *E. coli* homogenate:** Peak area of *S*-methyl captopril normalized to an internal standard (PAR) at two substrate concentrations for induced *E. coli* homogenate containing either full-length METTL7B (His-GST-METTL7B) or METTL7B incorporating a D98A point mutation (His-GST-METTL7B-D98A). Methyltransferase activity was measured using 5 mM captopril and 1 mg/mL total protein. Significant methyltransferase activity was only detected in homogenate containing His-GST-METTL7B. Data is presented as the mean ± s.d. Significance was determined using unpaired two-tailed *t* test. *****P*<0.0001.


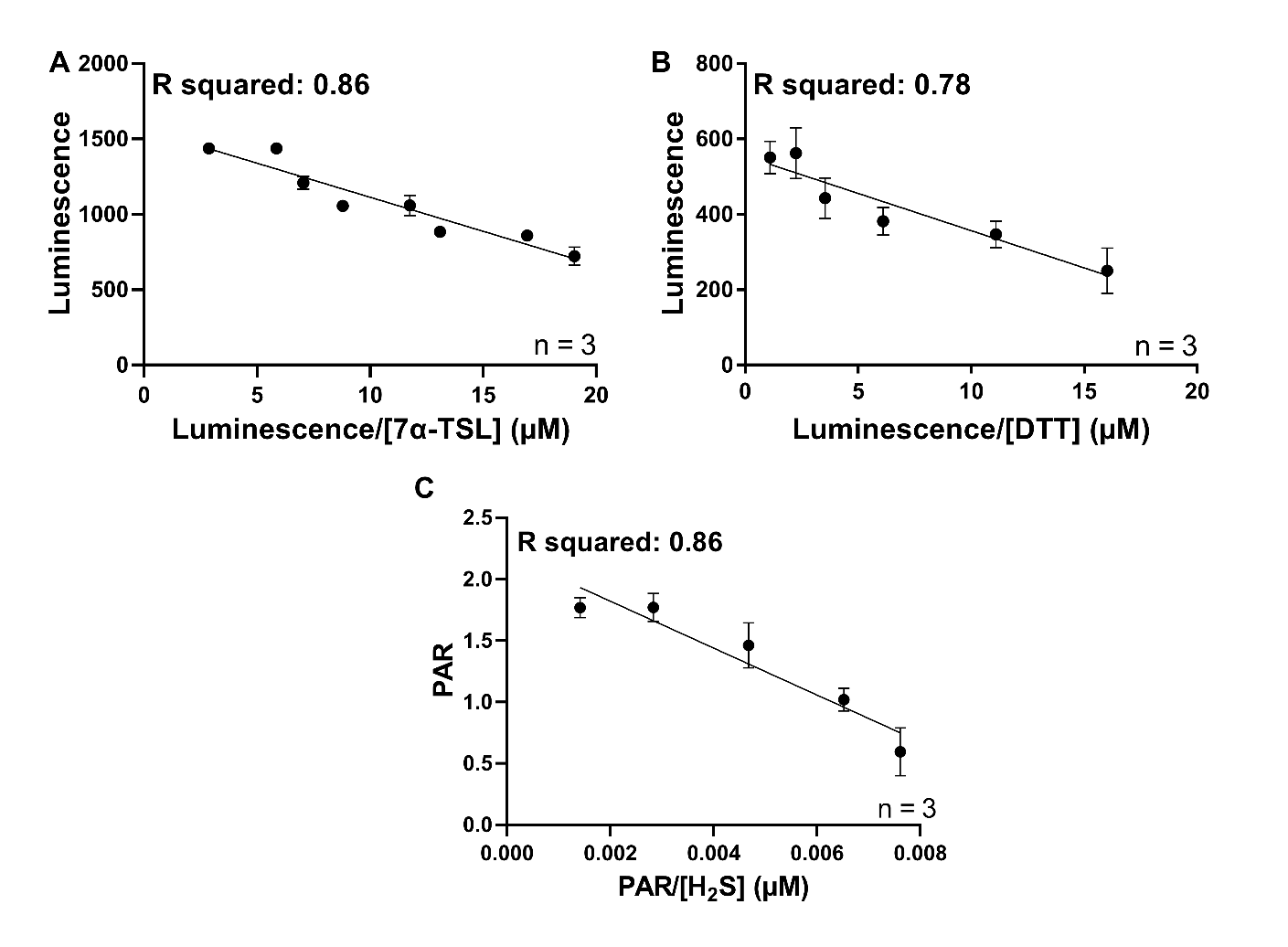


**Supplementary Figure 9: Eadie-Hofstee transformations of thiol methylation by His-GST-METTL7B. A)** Linearization of 7α-thiospironolactone methylation as measured by AdoHcy formation. **B)** Linearization of dithiothreitol methylation as measured by AdoHcy formation. **C)** Linearization of hydrogen sulfide methylation as measured by formation of methylsulfide. All data is presented as the mean ± standard deviation.
